# Does SARS-CoV-2 Affect Human Semen? A Systematic Review and Meta-Analysis

**DOI:** 10.1007/s10508-022-02520-3

**Published:** 2023-01-05

**Authors:** Tomasz Klepinowski, Marta Klepinowska, Leszek Sagan, Anhelli Syrenicz

**Affiliations:** 1 Department of Neurosurgery, Pomeranian Medical University Hospital No. 1, 71-252 Szczecin, Poland; 2 Department of Endocrinology, Metabolic and Internal Diseases, Pomeranian Medical University Hospital No. 1, Szczecin, Poland

**Keywords:** COVID-19, SARS-CoV-2, Coronavirus, Semen, Sperm, Sexual transmission

## Abstract

Contradictory results have been reported regarding effects of the SARS-CoV-2 upon human semen. A timely and up-to-date systematic review with meta-analysis appears necessary. This study aimed to deliver pooled prevalence (PP) of SARS-CoV-2 in semen and pooled semen parameters as compared with the uninfected. The relevant databases were scanned by two authors for observational studies reporting analysis of semen in COVID-19 patients. The SARS-CoV-2-infected were assigned to group A (exposed arm), whereas the uninfected to group B (unexposed arm). Newcastle–Ottawa Scale was used to address the risk of bias. PRISMA guidelines were adopted. In case of homogenous studies, fixed-effects model was followed, whereas for heterogenous studies random-effects model was used. Of 990 studies, 24 were eligible involving 1589 subjects (947 in group A and 642 in group B). The “comparability” domain was biased the most. SARS-CoV-2 RNA was detected in three studies among 8 individuals producing the PP of 1.76% (95% CI 0.72–3.21). Sperm concentration was reduced significantly (WMD = –16.23 [95% CI –25.56 to –6.89], as well as total sperm in ejaculate (WMD = –34.84 [95% CI − 43.51 to –26.17]) and sperm volume (WMD =  − 0.48 [95% CI − 0.59 to − 0.36] in group A as compared with controls. There was a non-significant effect upon progressive motility and leukocyte presence in semen. SARS-CoV-2 RNA in semen among the infected individuals is detected infrequently. By this token, sexual transmission through semen is of low probability and little concern for public health. However, significant decrease in sperm volume, sperm concentration, and total sperm in ejaculate has been noted. The current data, though, are limited, and more studies with longer follow-up are needed to evaluate the further impact.

**Supplementary Information:**

The online version contains supplementary material available at 10.1007/s10508-022-02520-3.

## Introduction

An unprecedented scale of the global pandemic caused by severe acute respiratory syndrome coronavirus 2 (SARS-CoV-2) has propelled researchers throughout the world to identify routes of its transmission. As the virus predominantly causes respiratory tract disease officially called coronavirus disease 2019 (COVID-19), it chiefly spreads through respiratory droplets during face-to-face contact with an infected subject. The disease, which until September 4, 2022, was declared in over 600 million cases with almost 6.5 million casualties worldwide (World Health Organization, https://covid19.who.int/), has also been of concern in terms of other organs and systems. By means of real-time reverse transcription polymerase chain reaction (rRT-PCR), viral nucleic acid was detected in nasal, nasopharyngeal, and pharyngeal smears, samples of saliva, blood, stool, urine, and tears (Karia et al., 2020; W. Wang et al., 2020). Regarding male reproductive tract, studies have been emerging focusing on possible presence of the viral RNA in semen and on the impact of COVID-19 upon semen characteristics (Cipriano et al., 2020; Holtmann et al., 2020; Temiz et al., 2021). Cell entry is facilitated by the viral spike protein and cellular angiotensin-converting enzyme 2 (ACE2) interaction (W. Li et al., 2003). Abundance of this enzyme in testes could possibly account for the viral appearance in semen and potentially another route of transmission. As new research has been conducted on the topic, an updated systematic review with meta-analysis is desired. Hence, the aim of this study is to perform the most comprehensive systematic review with statistical approach of the observational studies analyzing SARS-CoV-2 RNA in semen and sperm characteristics (sperm concentration, total sperm in ejaculate, volume, sperm motility, and presence of leukocytes). The following null hypotheses were stated: (1) 95% confidence intervals (CI) of weighted mean differences (WMD) of continuous variables encompasses 0 and 95% CI of risk ratio (RR) of binary variables includes 1 indicating SARS-CoV-2 and COVID-19 do not affect semen quality. (2) SARS-CoV-2 RNA in human semen is undetectable or its prevalence is negligible.

## Method

### Study Design and Search Strategy

It is a systematic review and meta-analysis of observational studies reporting analysis of semen in subjects diagnosed with COVID-19. An independent literature search by two authors was carried out on March 20, 2022. The scanned databases included PubMed MEDLINE, Scopus, EMBASE, and Web of Science. Full search strategy can be viewed as Electronic Supplementary Material 1. No temporal restrictions were imposed and all languages were acceptable. References of the eligible papers were given attention for additional articles. If data was incapable of being disaggregated, corresponding authors were consulted. For the systematic approach, transparency, and good research practice, we followed PRISMA guidelines (Electronic Supplementary Material 2).

### Inclusion Process

**Fig. 1:**
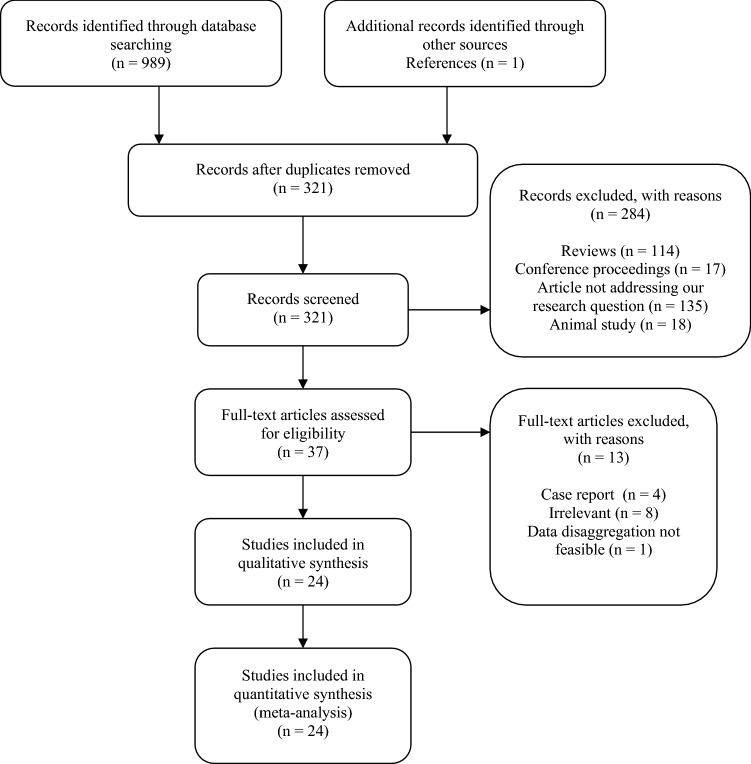
Study selection process

Eligibility criteria: (1) evaluate semen in COVID-19 patients (either in acute stage, in recovery stage, or both), (2) address only sexually mature subjects (set at 15 years of age), (3) and the number of subjects analyzed had to be equal to or greater than 5. Exclusion criteria: (1) animal studies, (2) reviews, (3) commentaries, (4) chapters, (5) conference proceedings, (6) posters, (7) surveys, (8) errata, (9) published in non-peer-reviewed supplements, (10) less than five participants, (11) high index of suspicion for the overlapping data, (12) data could not be disaggregated despite an attempt to consult with corresponding authors. Figure 1 depicts the inclusion process.

### Data Extraction and Curation

An attempt was made to extract the following data: (1) study design, (2) number of participants, (3) mean age, (4) mean/median follow-up, (5) number of controls, (6) geographical origin of the study, (7) number of patients with SARS-CoV-2 in semen, (8) laboratory method of SARS-CoV-2 confirmation, (9) interval between COVID-19 onset and semen collection, (10) semen parameters (sperm concentration, motility, volume, leukocyte count, bacterial presence, total sperm in ejaculate). Data were disaggregated into its components and then reaggregated for the statistical purpose. Missing means or standard deviations were calculated using the disaggregated data. If a study reported relevant parameters only as medians with interquartile ranges and there were strong hints of the overall distribution not deviating much from the normal distribution, then assumption would be made equaling median and mean as well as standard deviation (SD) = IQR/1.35. Microsoft Excel 2016 (Redmond, USA) was utilized for data curation. Participants were divided into two groups according to the infection status—group A consisting of SARS-CoV-2-positive (active or recovered) patients and group B comprising the uninfected.

### Risk of Bias and Quality

Risk of bias was addressed qualitatively and quantitatively. Qualitative assessment was grounded upon the Newcastle–Ottawa Scale (NOS), whereas for quantitative interpretation a funnel plot was schemed. For the NOS, the exposition was SARS-CoV-2 infection. The relevant endpoints were defined as presence of the viral RNA in semen and/or semen parameters as compared to World Health Organization reference values (5th centiles were adopted as the lower cut-off value) (Cooper et al., 2009). Eligible articles were assessed for three spectra: selection, comparability, and endpoints. Four stars could have been assigned for selection, two for comparability, and three for outcome, for “comparability” two stars, and for “outcome” three stars. Lack of any stars in a given domain meant the risk of bias was high. At least one star but shy of a maximum meant the risk of bias of moderate. A maximum number of stars meant the risk of bias was low. If there are no controls at all in a given study, then for the comparability domain as well as for ‘Selection of the non-exposed cohort’ part, it is assigned null. The most important control factor was age. Additional control factor was mean body mass index (BMI). As it was indicated that SARS-CoV-2 might affect semen even in the acute stage (D. Li et al., 2020a, 2020b), adequacy of minimal follow-up of cohorts (time from diagnosis to semen collection) was set at 0 days (the day of diagnosis).

### Statistical Analysis

Meta-analysis was conducted by means of MetaXL 5.3, EpiGear International Pty Ltd. (Brisbane, Australia) and Comprehensive Meta-Analysis V3 (Englewood, New Jersey, USA). Relative risk (risk ratio; RR) and weighted mean differences (WMD) were calculated with corresponding confidence intervals of 95%. Heterogeneity was evaluated based on *I*^2^ and chi^2^. Interpretation of *I*^2^ value was made as broadly accepted: 0–40%—homogenous; 40–60%—moderate heterogeneity; 60–80%—substantial heterogeneity; and 80–100%—considerable heterogeneity. Level of significance for Cochrane Q *p* value was arbitrarily set to < 10% (< 0.10), whereas level of significance for *p* value of comparative tests was universally set to < 5% (< 0.05). As in good research practice, in case of *I*^2^ ≥ 40% (heterogeneity), random-effects model would be adopted, whereas for *I*^2^ < 40% (homogeneity), fixed-effects model would be employed.

## Results

### Study Characteristics

A total of 989 results were identified at the first stage of database scanning and reference check yielded one additional paper. Of total 990 records, 321 remained after de-duplication. Further screening led to exclusion of 284 studies (Fig. 1). 37 full-text articles were checked for eligibility. Finally, 24 papers qualified, including one paper that had been collected in the reference check (Best et al., 2021; Burke et al., 2021; Donders et al., 2022; Erbay et al., 2021; Gacci et al., 2021; Guo et al., 2021; Hamarat et al., 2021; Holtmann et al., 2020; Kayaaslan et al., 2020; Koç & Keseroǧlu, 2021; D. Li et al., 2020a, 2020b; H. Li et al., 2020a, 2020b; Ma et al., 2021; Machado et al., 2021; Pan et al., 2020; Pavone et al., 2020; Pazir et al., 2021; Piroozmanesh et al., 2021; Rawlings et al., 2020; Ruan et al., 2021; Song et al., 2020; Temiz et al., 2021; M. Wang et al., 2022). 16 (66.7%) came from Asia (including six Turkish papers). 4 (16.7%) were conducted in Europe, and 4 (16.7%) in North America. The studies involved 1589 subjects that were divided into two groups: 947 in group A (59.6%; infected individuals) and 642 (40.4%) uninfected controls in group B. Mean age in group A was 35.6 years. Mean age in group B was 34.1 years. Mean BMI in group A was 24.0 kg/m^2^ whereas in group B 21.5 kg/m^2^. Semen specimens were collected at a mean time of 52 days from the disease onset (Table 1).

### Risk of Bias Assessment

Tabular display of Newcastle–Ottawa Scale is shown in Table 2. The comparability domain was biased the most. On the other hand, the outcome domain was characterized by the lowest risk of bias. Additional risk was found in the study of Temiz et al. as they had included three individuals with negative swab tests. Thus, a sensitivity analysis was conducted excluding their study from analysis.

**Table 1:** Study characteristics. RT-PCR—real-time polymerase chain reaction

1st author & year	Study design	Region	Method of systemic COVID-19 testing	No. of COVID-19 subjects (n)	Subjects with SARS-CoV-2 in semen
Wang 2022	Cohort	Asia	IgM, IgG	26	–
Donders 2021	Case series	Europe	RT-PCR	120	0
Machado 2021	Case series	North America	RT-PCR	15	1
Burke 2021	Case series	North America	RT-PCR	18	0
Piroozmanesh 2021	Cohort	Asia	RT-PCR and serum IgM, IgG	60	0
Best 2021	Case series	North America	RT-PCR	30	0
Koç 2021	Case series	Asia	RT-PCR	21	–
Pazir 2021	Cohort	Asia	RT-PCR	24	–
Erbay 2021	Cohort	Asia	RT-PCR	69	–
Rafiee 2021	Cohort	Asia	RT-PCR	200	–
Gacci 2021	Case series	Europe	RT-PCR	43	1
Hamarat 2021	Cohort	Asia	RT-PCR	41	–
Ma 2020	Case series	Asia	RT-PCR or serum IgM, IgG	12	0
Guo 2020	Case series	Asia	RT-PCR	23	0
Pavone 2020	Case series	Europe	RT-PCR	9	0
Ruan 2020	Cohort	Asia	RT-PCR	70	0
Holtmann 2020	Cohort	Europe	RT-PCR or serum IgA, IgG	20	0
Kayaaslan 2020	Case series	Asia	RT-PCR	16	0
Li D 2020	Case series	Asia	RT-PCR	38	6
Li H 2020	Cohort	Asia	RT-PCR	23	0
Pan 2020	Case series	Asia	qRT-PCR	34	0
Rawlings 2020	Case series	North America	dd-PCR	6	0
Song 2020	Case series	Asia	RT-PCR or serum IgM, IgG	12	0
Temiz 2020	Cohort	Asia	RT-PCR	17	0

*dd-PCR* Droplet digital polymerase chain reaction

**Table 2:** Qualitative assessment of risk of bias using Newcastle–Ottawa Scale

Study name	Selection	Comparability	Outcome
Donders 2022	✬ ✬	✬	✬ ✬ ✬
Machado 2021	✬ ✬		✬ ✬ ✬
Burke 2021	✬ ✬		✬ ✬ ✬
Piroozmanesh 2021	✬ ✬	✬ ✬	✬ ✬ ✬
Best 2021	✬ ✬	✬	✬ ✬ ✬
Koç 2021	✬ ✬ ✬	✬ ✬	✬ ✬
Pazir 2021	✬ ✬ ✬	✬ ✬	✬ ✬
Erbay 2021	✬ ✬ ✬	✬ ✬	✬ ✬
Rafiee 2021	✬ ✬ ✬	✬ ✬	✬ ✬
Gacci 2021	✬ ✬		✬ ✬ ✬
Hamarat 2021	✬ ✬ ✬	✬ ✬	✬ ✬
Ma 2020	✬ ✬		✬ ✬ ✬
Guo 2020	✬ ✬		✬ ✬ ✬
Pavone 2020	✬ ✬		✬ ✬ ✬
Ruan 2020	✬ ✬ ✬	✬	✬ ✬ ✬
Holtmann 2020	✬ ✬ ✬	✬	✬ ✬
Kayaaslan 2020	✬ ✬		✬ ✬ ✬
Li D 2020	✬		✬ ✬
Li H 2020	✬ ✬ ✬	✬	✬ ✬ ✬
Pan 2020	✬ ✬		✬ ✬ ✬
Rowlings 2020	✬ ✬		✬ ✬ ✬
Song 2020	✬ ✬		✬ ✬ ✬
Temiz 2020	✬ ✬ ✬	✬ ✬	✬ ✬ ✬

A total number of stars that could have been given for each domain was as follows: for “selection” four stars, for “comparability” two stars, and for “outcome” three stars

### Pooled Effects

**Fig. 2:**
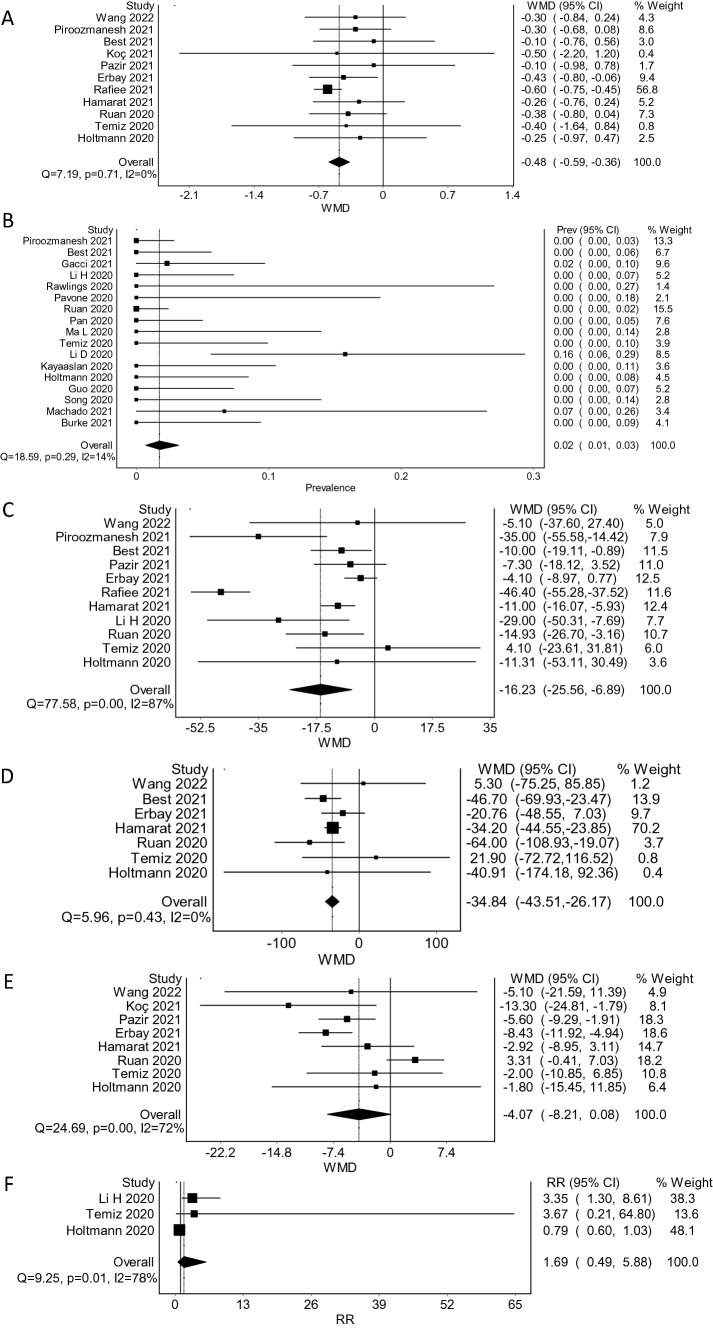
Forest plots of pooled size effects with corresponding 95% confidence intervals. **a** WMD in fixed-effects model for sperm volume; **b** Pooled prevalence in fixed-effects model of SARS-CoV-2 RNA in semen; **c** WMD in random-effects for sperm concentration; **d** WMD in fixed-effects model for total sperm per ejaculate; **e** random-effects model of WMD for progressive motility; **f** Random-effects model of RR for leukocytes in semen. RR—risk ratio. WMD—weighted mean difference

SARS-CoV-2 RNA was detected in semen at a pooled prevalence of 1.76% (95% CI 0.72–3.21; *I*^2^ = 14%, Cochran *Q* = 18.59, *p* = 0.29, fixed-effects model). Pooled sperm volume in group A was 2.64 ml versus 3.01 ml in group B. The weighted mean difference (WMD) for sperm volume was significant: − 0.48 (95% CI − 0.59 to − 0.37; *I*^2^ = 0%, Cochrane *Q* = 7.18, *p* = 0.71, fixed-effects model). Pooled prevalence of leukocytes in semen for group A was 50.57% and for group B 40.06% with non-significant risk ratio (RR) = 1.69 (95% CI 0.49–5.88; *I*^2^ = 78%, Cochran *Q* = 9.25, *p* = 0.01, random-effects model). Mean sperm concentration in the exposed was 43.97 [10^6^/mL], whereas in the unexposed 59.81 [10^6^/mL] with statistically significant WMD =  − 16.23 (95% CI − 25.56 to − 6.89; *I*^2^ = 87.1%, Cochran *Q* = 77.6, *p* < 0.01, random-effects model). Mean total sperm in ejaculate in group A was 108.41 [10^6^], whereas in group B 132.91 [10^6^] with statistically significant WMD =  − 34.84 (95% CI − 43.51 to − 26.17; *I*^2^ = 0%, Cochran *Q* = 5.96, *p* = 0.43, fixed-effects model). Mean progressive motility in group A was 32.49% (95% CI 38.58–44.18), whereas in group B it was 35.87% (95% CI 38.27–41.16) with non-significant WMD =  − 4.07 (95% CI − 8.21 to 0.08; *I*^2^ = 72%, Cochran *Q* = 24.69, *p* < 0.01, random-effects model). See Table 3 for the summary (Fig. 2).

**Table 3:** Summary of findings

Endpoint	Graphical illustration	Pooled effect size (95% CI)	No. of participants (studies)	Comment
Sperm volume	Fig. 2a	WMD − 0.35 (− 0.70 to 0.00)	1184 (11)	Significant decrease in sperm volume
SARS-CoV-2 RNA in semen	Fig. 2b	Prevalence 1.76% (0.72–3.21)	446 (17)	Three studies detected the viral RNA
Sperm concentration	Fig. 2c	WMD − 16.23 (− 25.56 to − 6.89)	1187 (11)	Significant decrease in sperm concentration [10^6^/mL]
Total sperm in ejaculate	Fig. 2d	WMD − 34.84 (− 43.51 to − 26.17)	594 (7)	Significant decrease in total sperm per ejaculate [10^6^]
Progressive motility	Fig. 2e	WMD -4.07 (− 8.21 to 0.08)	624 (8)	Non-significant effect on motility
Leukocytes in sperm	Fig. 2f	RR 1.69 (0.49–5.88)	107 (3)	Non-significant effect on leukocytes in semen

*CI* confidence interval. *RR* risk ratio. *WMD* weighted mean difference. *No.* number

### Sensitivity Analysis

In order to evaluate sperm quality specifically before and after COVID-19 in the same subjects, a separate analysis was conducted for studies that included longitudinal semen assessment. 7 of 24 papers included longitudinal cohorts (Best et al., 2021; Erbay et al., 2021; Hamarat et al., 2021; Koç & Keseroǧlu, 2021; Pazir et al., 2021; Rafiee & Tabei, 2021; M. Wang et al., 2022). Thus, the weighted mean differences were recalculated and compared with the primary results: (1) WMD for sperm concentration =  − 4.74 (95% CI − 27.08 to − 2.40, *Q* = 69.01, *p* < 0.01) remained significant. (2) WMD for sperm volume =  − 0.51 (95% CI − 0.64 to − 0.39, *Q* = 5.45, *p* = 0.49) remained significant. (3) WMD for total sperm per ejaculate =  − 34.17 (95% CI − 43.07 to − 25.28, *Q* = 2.93, *p* = 0.40) remained significant. (5) WMD for progressive motility =  − 6.71 (95% CI − 8.98 to − 4.44, *Q* = 4.09, *p* = 0.39) changed to significant.

## Discussion

### SARS-CoV-2 in Semen

Hereby, it has been confirmed that SARS-CoV-2 RNA, although rarely, might be present in semen. To date, 3 of 17 studies reported the RNA detection. Considering low pooled prevalence (1.76% [95% CI 0.72–3.21]), the findings of D. Li et al., Machado et al., and Gacci et al. appear incidental. Description of methodology of D. Li et al. and of Gacci et al. was scarce, as it did not provide technicalities about the procedure of semen collection or processing. Thus, site cross contamination might have been the case. Methodology of Machado et al. was more scrupulous, with subjects showering with soap just prior to masturbation and sample collection. Similarly, most of the studies that refuted the RNA presence in sperm, furnished detailed methodology describing careful washing of hands and penis with soap and water, drying it with paper towels, sterile containers, sterile transport system, as well as complex laboratory processing materials and methods (Guo et al., 2021; Kayaaslan et al., 2020; Song et al., 2020). By this token, the real pooled prevalence is expected be closer to the lower margin of the confidence interval. On the contrary, hypothetically the SARS-CoV-2 prevalence in semen be underestimated, since it has been indicated that PCR could be disturbed by various factors (Schrader et al., 2012). Regarding semen collection and processing, two are of concern: (1) powder from gloves and (2) urea from contamination by urine (Schrader et al., 2012; Tobe et al., 2017) and thereby could produce false negative results. Nonetheless, this updated systematic review with meta-analysis suggests the prevalence is low, and such is a risk of sexual transmission of SARS-CoV-2.

### Spermatogenesis Deregulation

The following semen characteristics were significantly affected in those who had been infected with SARS-CoV-2: total sperm per ejaculate, sperm concentration, and sperm volume. Although these parameters were reduced, when juxtaposed with norms of World Health Organization they were still above 5th centiles (Cooper et al., 2009). On one hand, febrile course of any disease might lead to transient spermatogenesis deregulation (Carlsen et al., 2003). Thus, there have been speculations whether fever could be the only culprit of the impaired spermatogenesis in COVID-19 (Bendayan & Boitrelle, 2020). On the other hand though, studies have shown that staying afebrile throughout the entire SARS-CoV-2 infection does not necessarily protect from deregulation of spermatogenesis (Donders et al., 2022; Holtmann et al., 2020). Disaggregation of the authors’ data to perform a subgroup meta-analysis of febrile versus afebrile was not feasible, thereby this matter remains unresolved. Interestingly, after having performed sensitivity analysis with studies of longitudinal semen evaluation, WMD of sperm progressive motility between two groups changed to statistically significant. This may reflect more heterogeneous nature of cross-sectional cohorts and more homogeneity of longitudinal cohorts.

### Impact of Other Viral Infections upon Semen

SARS-CoV-2 is not unique in having an impact on sperm quality. It has been also documented for human immunodeficiency virus (HIV) which may diminish ejaculate volume and progressive motility, although this might potentially be a side-effect of antiretroviral treatment (Goulart et al., 2020). The sperm parameters in AIDS patients could be correlated with CD4 leukocyte count: in a group with count greater than 350 cells/µl sperm vitality, sperm penetration, and sperm motility were significantly better than in those with CD4 count less than 350 cells/µl (D. Wang et al., 2014). In terms of other viruses, L1 capsid protein of human papillomavirus (HPV) binds to syndecan-1 in spermatozoa, which was shown to reduce progressive motility and increase a rate of antisperm antibodies (Garolla et al., 2013). Herpes simplex (HSV) infection usually does not affect sperm motility but was proven to reduce sperm count per ejaculate and seminal volume (Kurscheidt et al., 2018; Monavari et al., 2013). Furthermore, there might be an association between Epstein–Barr virus (EBV)-positive semen samples and leukocytospermia, without an impact upon sperm count or motility (Kaspersen & Höllsberg, 2013).

### Limitations

Although comprehensive, this meta-analysis is not without limitations. While some recent studies were of class II (Hamarat et al., 2021; Rafiee & Tabei, 2021; M. Wang et al., 2022), still majority of the literature on the effect of COVID-19 upon semen present evidence no stronger than class III. As authors are not unanimous in their expression of the semen parameters, not all of the characteristics could have been pooled (e.g., prevalence of bacteria in semen or febrile versus afebrile subgroup analysis). As identified through qualitative and quantitative risk of bias assessment, most studies might be biased, particularly in terms of comparability.

### Conclusions

Although SARS-CoV-2 has been detected in semen by several independent researchers, the pooled data suggest its prevalence is very low. Thus, semen as a serious transmission route is unlikely. Even though the viral RNA is usually undetectable in semen, COVID-19 alters its characteristics: sperm concentration, total sperm in ejaculate, and sperm volume are significantly reduced as compared to the uninfected. Progressive motility and prevalence of leukocytes in semen might remain unaffected. As more and more research is being conducted on the topic, this updated systematic review with meta-analysis pools more data and provides narrower confidence intervals and stronger evidence.

## Supplementary Information

Below is the link to the electronic supplementary material.

Supplementary file1 (DOCX 13 KB)

Supplementary file2 (DOC 65 KB)

## Data Availability

The data that support the findings of this study are available from the corresponding author, TK, upon reasonable request.
