# Supplementary material for: Does SARS-CoV-2 Affect Human Semen? A Systematic Review and Meta-Analysis
Source: Arch Sex Behav. 2023 Jan 5;52(2):669–77. doi: 10.1007/s10508-022-02520-3 (PMC9815676; doi:10.1007/s10508-022-02520-3)
Supplement: Supplementary file 1 — Supplementary file1 (DOCX 13 KB) [file 10508_2022_2520_MOESM1_ESM.docx]

Electronic Supplementary Material 1

**Full search strategy**

Search: **(((((SARS-CoV-2 AND sperm) OR (coronavirus AND sperm)) OR (COVID-19 AND sperm)) OR (COVID-19 AND semen)) OR (coronavirus AND semen)) OR (SARS-CoV-2 AND semen)**

(("sars cov 2"[MeSH Terms] OR "sars cov 2"[All Fields] OR "sars cov 2"[All Fields]) AND ("sperm s"[All Fields] OR "spermatozoa"[MeSH Terms] OR "spermatozoa"[All Fields] OR "sperm"[All Fields] OR "sperms"[All Fields])) OR (("coronavirus"[MeSH Terms] OR "coronavirus"[All Fields] OR "coronaviruses"[All Fields]) AND ("sperm s"[All Fields] OR "spermatozoa"[MeSH Terms] OR "spermatozoa"[All Fields] OR "sperm"[All Fields] OR "sperms"[All Fields])) OR (("severe acute respiratory syndrome coronavirus 2"[Supplementary Concept] OR "severe acute respiratory syndrome coronavirus 2"[All Fields] OR "ncov"[All Fields] OR "2019 ncov"[All Fields] OR "covid 19"[All Fields] OR "sars cov 2"[All Fields] OR (("coronavirus"[All Fields] OR "cov"[All Fields]) AND 2019/11/01:3000/12/31[Date - Publication])) AND ("sperm s"[All Fields] OR "spermatozoa"[MeSH Terms] OR "spermatozoa"[All Fields] OR "sperm"[All Fields] OR "sperms"[All Fields])) OR (("severe acute respiratory syndrome coronavirus 2"[Supplementary Concept] OR "severe acute respiratory syndrome coronavirus 2"[All Fields] OR "ncov"[All Fields] OR "2019 ncov"[All Fields] OR "covid 19"[All Fields] OR "sars cov 2"[All Fields] OR (("coronavirus"[All Fields] OR "cov"[All Fields]) AND 2019/11/01:3000/12/31[Date - Publication])) AND ("semen"[MeSH Terms] OR "semen"[All Fields] OR "semen s"[All Fields] OR "semens"[All Fields])) OR (("coronavirus"[MeSH Terms] OR "coronavirus"[All Fields] OR "coronaviruses"[All Fields]) AND ("semen"[MeSH Terms] OR "semen"[All Fields] OR "semen s"[All Fields] OR "semens"[All Fields])) OR (("sars cov 2"[MeSH Terms] OR "sars cov 2"[All Fields] OR "sars cov 2"[All Fields]) AND ("semen"[MeSH Terms] OR "semen"[All Fields] OR "semen s"[All Fields] OR "semens"[All Fields]))

**Translations**

**SARS-CoV-2:** "sars-cov-2"[MeSH Terms] OR "sars-cov-2"[All Fields] OR "sars cov 2"[All Fields]

**sperm:** "sperm's"[All Fields] OR "spermatozoa"[MeSH Terms] OR "spermatozoa"[All Fields] OR "sperm"[All Fields] OR "sperms"[All Fields]

**coronavirus:** "coronavirus"[MeSH Terms] OR "coronavirus"[All Fields] OR "coronaviruses"[All Fields]

**sperm:** "sperm's"[All Fields] OR "spermatozoa"[MeSH Terms] OR "spermatozoa"[All Fields] OR "sperm"[All Fields] OR "sperms"[All Fields]

**COVID-19:** "severe acute respiratory syndrome coronavirus 2"[Supplementary Concept] OR "severe acute respiratory syndrome coronavirus 2"[All Fields] OR "ncov"[All Fields] OR "2019-nCoV"[All Fields] OR "COVID-19"[All Fields] OR "SARS-CoV-2"[All Fields] OR ((coronavirus[All Fields] OR "cov"[All Fields]) AND 2019/11:3000[pdat])

**sperm:** "sperm's"[All Fields] OR "spermatozoa"[MeSH Terms] OR "spermatozoa"[All Fields] OR "sperm"[All Fields] OR "sperms"[All Fields]

**COVID-19:** "severe acute respiratory syndrome coronavirus 2"[Supplementary Concept] OR "severe acute respiratory syndrome coronavirus 2"[All Fields] OR "ncov"[All Fields] OR "2019-nCoV"[All Fields] OR "COVID-19"[All Fields] OR "SARS-CoV-2"[All Fields] OR ((coronavirus[All Fields] OR "cov"[All Fields]) AND 2019/11:3000[pdat])

**semen:** "semen"[MeSH Terms] OR "semen"[All Fields] OR "semen's"[All Fields] OR "semens"[All Fields]

**coronavirus:** "coronavirus"[MeSH Terms] OR "coronavirus"[All Fields] OR "coronaviruses"[All Fields]

**semen:** "semen"[MeSH Terms] OR "semen"[All Fields] OR "semen's"[All Fields] OR "semens"[All Fields]

**SARS-CoV-2:** "sars-cov-2"[MeSH Terms] OR "sars-cov-2"[All Fields] OR "sars cov 2"[All Fields]

**semen:** "semen"[MeSH Terms] OR "semen"[All Fields] OR "semen's"[All Fields] OR "semens"[All Fields]
